# DKK1 Promotes Epithelial–Mesenchymal Transition and Cisplatin Resistance in Gastric Cancer via Activation of the PI3K/AKT Pathway

**DOI:** 10.3390/cancers15194756

**Published:** 2023-09-27

**Authors:** Jian Li, Yaqiong Zhang, Fangzhou Ye, Peiyu Qian, Zhe Qin, Deming Li, Li Ye, Li Feng

**Affiliations:** 1 Endoscopy Center, Minhang Hospital, Fudan University, Shanghai 201199, China; jianli21@m.fudan.edu.cn (J.L.); 21111360003@m.fudan.edu.cn (Y.Z.); 23111360002@m.fudan.edu.cn (F.Y.); 22111360005@m.fudan.edu.cn (Z.Q.); 22111360004@m.fudan.edu.cn (D.L.); 2 Institutes of Biomedical Sciences, Fudan University, Shanghai 200032, China; 19231510043@fudan.edu.cn; 3 School of Pharmacy and State Key Laboratory of Quality Research in Chinese Medicine, Macau University of Science and Technology, Macau SAR 999078, China

**Keywords:** Dickkopf-related protein 1, gastric cancer, cancer therapeutic resistance, epithelial-to-mesenchymal transition

## Abstract

**Simple Summary:**

Dickkopf-related protein 1 (DKK1) plays different roles in different cancers, and its aberrant expression is associated with tumor progression and poor prognosis. However, the role of DKK1 in gastric cancer is not well studied or understood, especially regarding its function in gastric cancer chemoresistance. Chemotherapy is a fundamental treatment method for gastric cancer. Resistance to chemotherapy limits chemotherapeutic agents in clinical applications, and the mechanism of drug resistance needs to be elucidated. In this study, we found that DKK1 was highly expressed in gastric cancer patients and cisplatin (CDDP)-resistant cell lines. DKK1 was able to activate the PI3K/AKT pathway and affect epithelial-to-mesenchymal transition (EMT), further contributing to CDDP resistance. DKK1 inhibition recovered CDDP sensitivity both in vitro and in vivo. Therefore, our study highlights the potential targeted inhibition of DKK1 to reverse CDDP resistance and alleviate metastatic properties in gastric cancer.

**Abstract:**

Chemotherapy is a classical method of cancer treatment. Cisplatin-based chemotherapy is a traditional and essential therapeutic approach in gastric cancer treatment. However, the development of drug resistance during treatment is a major obstacle that limits their further application, and molecular changes have occurred in the development of drug resistance. Here, we found that Dickkopf-related protein 1 (DKK1) is highly expressed in gastric cancer and related to poor prognosis in gastric cancer patients through public database mining. Next, we also identified that DKK1 is highly expressed in CDDP-resistant gastric cancer cell lines, supporting the notion that DKK1 is a necessary regulator of CDDP resistance. In terms of mechanistic research, our data reveal that DKK1 was able to activate the PI3K/AKT pathway and affect epithelial-to-mesenchymal transition, further contributing to CDDP resistance. Genetic knockdown and pharmacological inhibition of DKK1 recovered CDDP sensitivity both in vitro and in vivo. Therefore, our study highlights the potential of targeted inhibition of DKK1 to reverse CDDP resistance and alleviate metastatic properties in gastric cancer.

## 1. Introduction

Gastric cancer (GC) is a common malignancy and a major cause of cancer-related deaths worldwide. It was estimated that there were 1.03 million new cases and 769,000 deaths from gastric cancer in 2020 [1]. The incidence and mortality rates of gastric cancer vary greatly by geographic region, with the highest rates reported in Eastern Asia. Endoscopic therapy is the first choice for early-stage gastric cancer. The treatment for advanced gastric cancer usually centers around chemotherapy and targeted therapy [2]. Cisplatin (CDDP)-based combination chemotherapy regimens are the first-choice treatment for advanced gastric cancer patients recommended by the Chinese Society of Clinical Oncology (CSCO) gastric cancer diagnosis and treatment guidelines [3]. The mechanism of CDDP is that CDDP inhibits DNA replication and transcription via Pt/DNA complexes, further leading to cell death [4]. However, many patients are sensitive to CDDP in the initial stages of chemotherapy and gradually develop CDDP resistance [5,6]. Once the drug resistance develops, treatment options become limited, making the treatment of advanced gastric cancer challenging. CDDP resistance is a multigenic, multifactorial process that includes reduced drug entry into cells and increased efflux, increased chelation and detoxification of tumor cells, enhanced platinum-induced DNA damage repair and tolerance, key pathway activation, metabolic reprogramming, and changes in the tumor microenvironment [7]. As a result, exploring new targets to overcome CDDP resistance and improve treatment outcomes for gastric cancer patients remains an ongoing challenge and an area of active research.

Dickkopf-related protein 1 (DKK-1) is a member of the DKK gene family, consisting of a signal peptide sequence and two regions rich in cysteine. DKK1 was first identified as a head inducer during embryonic development and later found to act as an antagonist of the Wnt signaling pathway [8]. Accumulating evidence shows that dysregulation of DKK1 is involved in tumor development and progression [9,10,11,12]. For instance, Tao et al. found that DKK1 was highly expressed in human non-small cell lung cancer (NSCLC) samples and closely associated with vasculogenic mimicry, which contributed to greater invasiveness of cancer cells [10]. Elevated DKK1 expression was also observed in prostate cancer, and it could be an indicator of poor survival in prostate cancer patients [13]. In contrast, DKK1 was downregulated in colorectal cancer (CRC) and participated in the suppression of CRC tumorigenesis and angiogenesis [14], acting as a cancer suppressor gene. In addition, DKK1 activation in melanoma gives rise to apoptosis in vivo, and DKK1 also plays a suppressor role in melanoma [15]. In terms of drug resistance, the serum level of DKK1 in the malignant group was higher than that in the benign group and healthy control group. Interestingly, the serum DKK1 level in the drug-resistant group was higher than that in the sensitive group, suggesting that DKK1 can be used as a marker for lung cancer screening, diagnosis, and chemotherapy efficacy evaluation [16]. Moreover, DKK1 is also highly expressed in CDDP-resistant cells in non-small cell lung cancer and ovarian cancer. DKK1 plays a role in driving the CDDP-resistant phenotype, and knocking down DKK1 can make NSCLC cells and ovarian cancer cells sensitive to CDDP [17]. However, the role of DKK1 in GC development and CDDP resistance remains unclear. 

Epithelial–mesenchymal transition (EMT) is a cellular process characterized by the transformation of epithelial cells into mesenchymal cells. In this process, epithelial cells gain migration and motor capacity while losing cell polarity under the stimulation of various extracellular factors [18]. Tumor cells are more likely to undergo EMT, which is manifested by the upregulation of mesenchymal markers, including N-cadherin [19] and vimentin, and the downregulation of epithelial markers, including E-cadherin [20]. Hence, EMT is an underlying mechanism that enables tumor cells to obtain metastatic features. In addition, EMT is also involved in tumor cell drug resistance. Research from Korea reported that through large-scale cancer cell transcriptome and related drug response dataset mining, the authors found that EMT is a common cause of chemotherapy resistance [21]. In addition, a recent study published in Nature revealed that EMT cancer cells are highly insensitive to anticancer approaches both in vitro and in vivo [22]. Therefore, EMT plays an important role in tumor metastasis and drug resistance. 

In this study, we first found that DKK1 was highly expressed in GC tissue and that its expression was related to poor survival. Second, through western blotting, we found that DKK1 was upregulated in CDDP-resistant GC cells. We aimed to explore the role and molecular mechanism of the DKK1-PI3K/AKT-EMT axis in sensitizing GC cells to CDDP and evaluate the efficacy of targeting DKK1. Third, to provide an experimental foundation for a solution to overcome GC drug resistance, we combined the anti-DKK1 Ab (mDKK1) with CDDP as a potential therapy method to reverse drug resistance in vitro and in vivo.

## 2. Materials and Methods

### 2.1. Drugs and Reagents

Cisplatin (cat. no. HY-17394) and PI3K/AKT inhibitor (cat. no. HY-10108) were purchased from MedChemExpress. The antibodies used for western blot and immunohistochemistry were as follows: anti-DKK1 antibody (cat. no. ab307367), anti-Phospho-PI3K antibody (cat. no. 4228) were purchased from Abcam (Cambridge, UK) and Cell Signaling Technology (Danvers, MA, USA); anti-Ki67 antibody (cat. no. ab15580) was purchased from Abcam; anti-vimentin antibody (cat. no. 60330-1-lg), anti-N-cadherin antibody (cat. no. 22018-1-AP), anti-E-cadherin antibody (cat. no. 60335-1-lg), anti-GAPDH antibody (cat. no. 60004-1-Ig), anti-PI3K antibody (cat. no. 60225-1-Ig), anti-AKT antibody (cat. no. 60203-2-Ig), anti-Phospho-AKT (cat. no. 28731-1-AP) were purchased from Proteintech Group (Rosemont, IL, USA). HRP-linked secondary antibody anti-rabbit lgG (cat. no. 7074) and anti-mouse lgG (cat. no. 7076) were obtained from Cell Signaling Technology. 

### 2.2. Cell lines and Cell Culture

The human gastric cancer cell lines GES-1, SGC7901, MGC803, HGC27, HGC27/DDP, and SGC7901/DDP were obtained from the Shanghai Institutes for Biological Sciences, Chinese Academy of Sciences Cell Bank (Shanghai, China) and cultured in RPMI-1640 medium supplemented with 10% fetal bovine serum (FBS) and 1% penicillin–streptomycin in a humidified atmosphere containing 5% CO_2_ at 37 °C. HGC27/DDP and SGC7901/DDP cells were cultured in RPMI-1640 medium supplemented with 2–4 μM CDDP, 10% fetal bovine serum (FBS), and 1% penicillin–streptomycin in a humidified atmosphere containing 5% CO_2_ at 37 °C. Cells were passaged every 2–3 days using trypsin-EDTA (0.25%) and then subcultured at a 1:3 ratio.

### 2.3. Western Blot

Western blotting was performed to detect the expression levels of proteins. Briefly, cells were lysed with RIPA buffer (Beyotime, Shanghai, China) containing a protease inhibitor cocktail (Cell Signaling Technology). The protein concentration was measured using a BCA protein assay kit (Beyotime, Shanghai, China). Equal amounts of protein were separated by SDS-PAGE and transferred to a nitrocellulose membrane (Millipore, Bedford, MA, USA). After blocking with 5% nonfat milk, the membrane was incubated with primary antibodies against the target proteins overnight at 4 °C, followed by HRP-conjugated secondary antibodies for 2 h at room temperature. The bands were visualized using an enhanced chemiluminescence detection system (Bio-Rad, Hercules, CA, USA) and analyzed with ImageJ software version 1.54f (NIH, Bethesda, MD, USA). GAPDH was used as an internal control. ImageJ software version 1.54f was used to analyze the relative levels of protein expression. The original western blot figures could be found in the Supplementary Materials.

### 2.4. Vector Construction and Transfection

The plasmid vector used in this study was constructed as follows. The target DKK1 gene was amplified by PCR from a cDNA template, Flag (3× Flag)-tagged DKK1 was cloned and inserted into the pLVX plasmid, and three shRNAs synthesized by Hanbio Tech (Shanghai, China) were cloned and inserted into the pLVX plasmid. HEK293T cells were used in this study for lentivirus packaging. pLVX-DKK1 or pLVX-DKK1-shRNAs were then cotransfected with lentivirus packaging plasmids psPAX2 (Addgene plasmid, 12260) and pMD2. G (Addgene plasmid, 12259) via Lipofectamine^®^ 2000 (Invitrogen, Waltham, MA, USA). For transfection, cells were seeded into 6-well plates and allowed to reach 80–90% confluence. Viruses were then harvested at 48 h and 72 h after transfection. To establish stable cell lines, transfected cells, and negative control viruses were selected with puromycin (2 μg/mL, Solarbio, Beijing, China) until resistant colonies were visible. These colonies were then expanded and screened for the expression of DKK1 by western blotting.

### 2.5. Colony Formation Assay

Cells were seeded into 6-well plates at a density of 500 cells per well and cultured for 10–14 days until visible colonies formed. The colonies were then fixed with 4% paraformaldehyde and stained with crystal violet. The number of colonies containing at least 50 cells was counted under a microscope. Each experiment was repeated three times to ensure reproducibility of the results.

### 2.6. CCK-8 Assay

Cells were digested with trypsin and then diluted to a density of 5 × 10^4^ cells/mL. Then, 100 μL of the cell suspension was cultured in each well of a 96-well culture plate, and 3 replicate wells were set up. The cell density for each well was 5 × 10^3^ cells/well, and 100 μL culture medium was added as a blank control. Different drug concentrations were added to the indicated cell groups. After treatment for 48 h, mix Cell Counting Kit-8 (CCK-8) and serum-free essential medium were mixed at a volume ratio of 1:10. Then, 110 μL of the mixture was added to each well and incubated at 37 °C and 5% CO_2_ for 3–4 h. A microplate reader was used to measure the absorbance at 450 nm. The percentages of viable cells were calculated, and dose-response curves were plotted.

### 2.7. Flow Cytometry

Flow cytometry was used to detect apoptotic cells. Briefly, cells were collected, washed with PBS after different treatments, and then resuspended in binding buffer at a concentration of 1 × 10^6^/mL. Then, 5 μL Annexin V-FITC and 5 μL propidium iodide (PI) were added to the cell suspension and incubated for 15 min at room temperature. After washing, the cells were analyzed on a flow cytometer (BD Biosciences, San Jose, CA, USA). The results were analyzed using FlowJo software version 10.9 (Tree Star, Ashland, OR, USA). The percentage of apoptotic cells was determined by measuring the Annexin V-FITC-positive and PI-negative cells.

### 2.8. Wound Scratch and Transwell Assays

Wound scratch and transwell assays were performed to evaluate cell migration and invasion, respectively. For the wound scratch assay, cells were seeded in a 6-well culture plate and allowed to grow to confluence. The cell monolayer was scratched with a sterile pipette tip and washed with PBS to remove the debris. The cells were then incubated in serum-free medium for 24 h, and wound closure was observed and photographed under an inverted microscope (Olympus, Tokyo, Japan). For the transwell assay, cells were seeded in the upper chamber of a transwell insert (Corning Inc., Corning, NY, USA) with or without Matrigel (BD Biosciences, San Jose, CA, USA) coating. The lower chamber was filled with medium containing 10% FBS as a chemoattractant. After 24 h of incubation, the cells that migrated or invaded through the membrane were fixed with 4% paraformaldehyde and stained with crystal violet. The cells were then observed and photographed under an inverted microscope (Olympus, Tokyo, Japan).

### 2.9. Xenograft Tumors in Nude Mice

The xenograft tumor model was established by subcutaneously injecting 5 × 10^6^ SGC7901/DDP tumor cells in 100 µL of PBS into the right flank of 6-week-old female nude mice (BALB/c). The mice were randomly divided into different treatment groups (n = 6/group) when the tumors reached a volume of approximately 100 mm^3^. The treatment groups received intraperitoneal injections of the drug or vehicle control once a day for 21 consecutive days. Tumor volume was measured every 3 days using a caliper, and tumor volume was calculated as (length × width^2^)/2. The mice were euthanized by CO_2_ inhalation at the end of the experiment, and tumors were excised and weighed. The tumor growth inhibition rate was calculated as (1 − average tumor weight in the treatment group/average tumor weight in the control group) × 100%.

### 2.10. Immunohistochemistry

Immunohistochemistry (IHC) assays were performed to detect the protein expression of target molecules in tumor tissues. Briefly, paraffin-embedded tissue sections were deparaffinized, rehydrated, and subjected to antigen retrieval using citrate buffer (pH 6.0) in a microwave oven. Endogenous peroxidase activity was blocked with 3% H_2_O_2_, and nonspecific binding was blocked with 5% goat serum. The sections were incubated with primary antibodies against the target molecules overnight at 4 °C and then with HRP-conjugated secondary antibodies for 1 h at room temperature. The sections were stained with 3,3’-diaminobenzidine (DAB) and counterstained with hematoxylin. The images were observed and photographed under an optical microscope (Olympus, Tokyo, Japan). The staining intensity and percentage of positive cells were evaluated by two independent pathologists who were blinded to the clinical information.

### 2.11. Bioinformatical Analysis

The DKK1 expression profile data for different cancers were obtained from TIMER (http://timer.comp-genomics.org/timer/ (accessed on 3 March 2022)), and survival data were obtained from GEPIA (Gene Expression Profiling Interactive Analysis; http://gepia.cancer-pku.cn/ (accessed on 3 March 2022)). TIMER is a comprehensive resource for systematic analysis of different cancers. The entire database incorporates 1065 gastric carcinoma samples and corresponding gene expression data. A gene differential expression module was used to study the differential expression between tumor and adjacent normal tissues for DKK1. The statistical significance was computed by the Wilcoxon test. Low and high DKK1 expression in the RNA-seq data was defined as *p* adjust < 0.05, |Log2FC| ≥ 1. The overall survival data and disease-free survival data in our study were from GEPIA. We selected the median as the expression threshold for splitting the high-expression and low-expression cohorts. The hazard ratio was calculated based on the Cox PH model. Cutoff-High (50%, default setting by median): samples with expression levels higher than this threshold are considered as the high-expression cohort. Cutoff-Low (50%, default setting by median): samples with expression levels lower than this threshold are considered the low-expression cohort.

### 2.12. Immunofluorescence (IF)

Cells were grown on glass coverslips and fixed in 4% paraformaldehyde for 20 min at room temperature. After washing with PBS, the cells were permeabilized with 0.1% Triton-X for 5 min and then blocked with 5% BSA for 1 h. Primary antibody against Ki67 (1:500) was added and incubated overnight at 4 °C. Cells were then washed and incubated with secondary antibodies conjugated to Alexa Fluor 488 or 594 (1:500) for 1 h at room temperature. Finally, the cells were stained with DAPI and mounted on glass slides. Images were obtained under a confocal microscope (Leica Microsystems, Milton Keynes, UK).

### 2.13. Short Hairpin RNA (shRNA) Transfection

Gastric cancer cells were transfected with shRNA constructs targeting the expression of the gene of interest using Lipofectamine 2000 (Invitrogen) according to the manufacturer’s instructions. Briefly, cells were seeded in 6-well plates at a density of 1 × 10^5^ cells per well and allowed to attach overnight. The shRNA plasmids were mixed with Lipofectamine 2000 reagent in Opti-MEM medium and incubated for 20 min at room temperature. The mixture was then added to the cells and incubated for 24 h at 37 °C in a CO_2_ incubator. Cells were then selected with puromycin (2 μg/mL) for 48 h to obtain stable knockdown cell lines. The efficiency of shRNA knockdown was evaluated by qRT-PCR and Western blotting. 

The shRNA sequences used in this study were as follows:

- shRNA1: 5′-CCTGTCCTGAAAGAAGGTCAA-3′
- shRNA2: 5′-CCAGAAGAACCACCTTGTCTT-3′
- shRNA3: 5′-CCTGTCCTGAAAGAAGGTCAA-3′

A non-targeting shRNA construct was used as a negative control. The sequences of the non-targeting shRNA were as follows:

- shRNA-NC: 5′-TTCTCCGAACGTGTCACGTAA-3′

### 2.14. RNA-Sequencing

Total RNA was extracted from shNC and shDKK1 GC cells using the Qiagen RNeasy Kit. RNA quality and quantity were assessed using an Agilent 2100 Bioanalyzer. RNA libraries were constructed using the Illumina TruSeq RNA Library Prep Kit, following the manufacturer’s protocol. Sequencing was performed on the Illumina Novaseq 6000 to generate 150 bp paired-end reads. Gene set enrichment analysis (GSEA) was performed using GSEA software (version 4.3.2). The significance of enrichment for each pathway was evaluated based on a hypergeometric test, with an adjusted *p*-value < 0.05 deemed significant. Kyoto Encyclopedia of Genes and Genomes (KEGG) pathway enrichment analysis was visualized via Cytoscape software (version 3.7.2).

### 2.15. Statistical Analysis

Statistical analysis was performed using SPSS software (version 22.0, IBM Corp., Armonk, NY, USA). The data are presented as the mean ± standard deviation (SD) from at least three independent experiments. The statistical significance of differences was determined by Student’s *t*-test or one-way analysis of variance (ANOVA) followed by Tukey’s post hoc test. *p* values < 0.05 were considered statistically significant.

## 3. Results

### 3.1. DKK1 Is Highly Expressed in GC Tissues and Associated with Poor Prognosis in GC Patients

**Figure 1:**
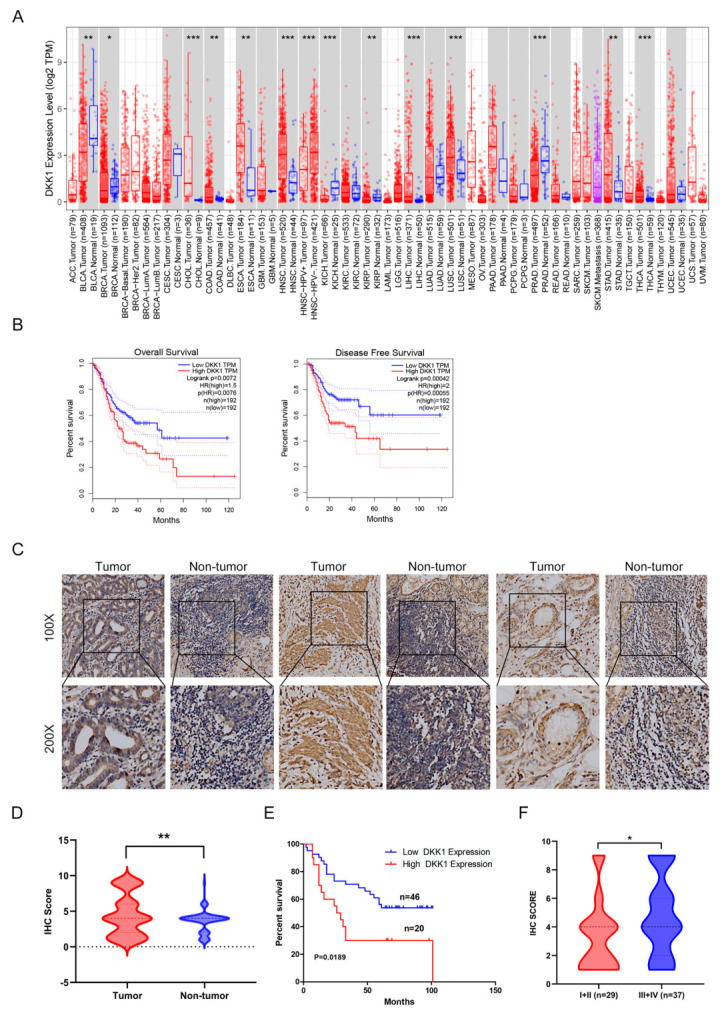
DKK1 is highly expressed in GC tissues and associated with poor prognosis in GC patients. (**A**) DKK1 transcription profiles of different cancers in the GEPIA dataset. Compared to normal tissue, the DKK1 transcriptional level was significantly higher in GC tissues. Distributions of DKK1 transcriptional levels in tumor and normal tissues are displayed using box plots. Red: DKK1 expression in tumor tissue, blue: DKK1 expression in normal tissue. The statistical significance was computed by the Wilcoxon test. * *p* < 0.05, ** *p* < 0.01, *** *p* < 0.001. (**B**) High DKK1 expression was associated with poor OS (*p* = 0.0072) and DFS (*p* = 0.00042) in GC. (**C**,**D**) Tissue microarray showed that DKK1 was significantly highly expressed in GC tissue samples compared with adjacent normal tissues. Scale bar: 50 μm. A violin plot was used to show DKK1 protein expression levels by immunohistochemical staining in 66 pairs of clinical GC samples. ** *p* < 0.01 using a 2−tailed paired Student’s *t*-test. (**E**) Survival data from the tissue microarray revealed that high DKK1 expression was associated with poor survival and may become a biomarker in GC patients. *p* is based on a log-rank test, *p* = 0.0189. (**F**) DKK1 expression status in early (TNM I + II) and advanced (TNM III + IV) stage GC. *p* is based on a log-rank test. * *p* < 0.05.

To characterize the DKK1 expression level in tumor tissue and adjacent nontumor tissue. A public database was used to analyze DKK1 expression levels in different cancers, as shown in Figure 1A. Compared to the corresponding nontumor tissues, DKK1 was significantly highly expressed in bladder urothelial carcinoma (BLCA), cholangiocarcinoma (CHOL), colon adenocarcinoma (COAD), esophageal carcinoma (ESCA), head and neck squamous cell carcinoma (HNSC), kidney chromophobe (KICH), kidney renal papillary cell carcinoma (KIRP), liver hepatocellular carcinoma (LIHC), lung squamous cell carcinoma (LUSC), prostate adenocarcinoma (PRAD), stomach adenocarcinoma (STAD) and thyroid carcinoma (THCA) (** *p* < 0.01, *** *p* < 0.001). Moreover, high DKK1 mRNA transcripts were closely related to poor overall survival (OS) and disease-free survival (DFS) in gastric cancer, *p* = 0.0072 and *p* = 0.00042 (Figure 1B).

To further identify DKK1 expression in GC tissues, we evaluated DKK1 protein levels via a human GC tissue microarray (STC1601) from SUPERBIOTEK (Shanghai, China). Compared with adjacent nontumor tissue, DKK1 was highly expressed in GC tissue (Figure 1C,D). Consistent with the data from the database, the Kaplan–Meier survival curve was drawn from the GC tissue microarray, and it was also revealed that DKK1 could serve as an indicator of worse prognosis and advanced stages in GC patients, *p* < 0.05 (Figure 1E,F). Through multivariate Cox analysis, we found that tumor size and TNM stage were associated with the survival of GC patients (Table 1). In univariate COX regression analysis, our data showed that T stage, N stage, TNM stage, and tumor size were the significant risk factors affecting GC patient survival (*p <* 0.05) (Table 2). In summary, the above results showed that DKK1 was highly expressed in GC cancer and was related to higher tumor stage and poor prognosis.

### 3.2. Characterization of Cisplatin Resistant Gastric Cancer Cell Lines

**Figure 2:**
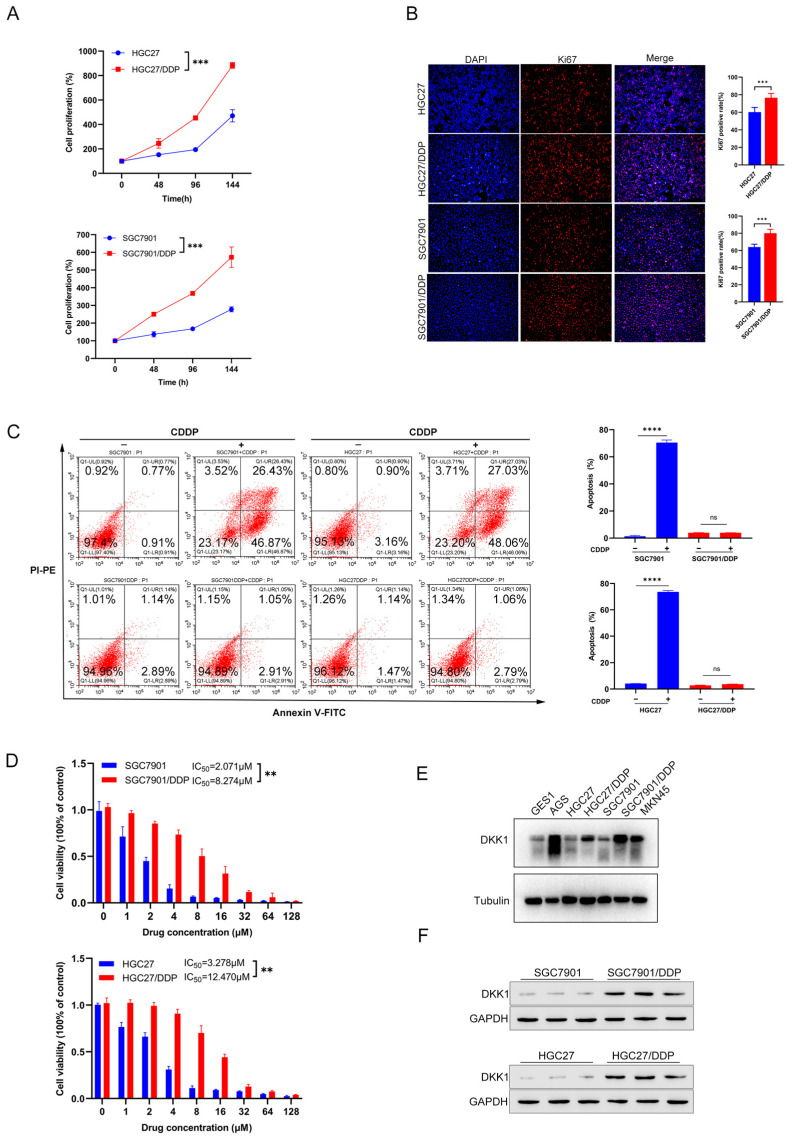
Characterization of CDDP sensitive and resistant GC cell lines. (**A**) Comparison of cell proliferation via CKK8 assay in CDDP-sensitive and CDDP-resistant cell lines over 144 h. Data were from independent experiments. Two-way ANOVA was used. (**B**) Immunofluorescence staining of Ki67 in HGC27, SGC7901, HGC27/DDP and SGC7901/DDP cells. Representative images were obtained by microscopy. Scale bar: 100 μm. (**C**) Flow cytometry assay of apoptotic cells in CDDP-resistant and corresponding CDDP-sensitive cells under the CDDP treatment. (**D**) Cell viability assay of HGC27, HGC27/DDP, SGC7901 and SGC7901/DDP under the CDDP treatment. (**E**) DKK1 protein expression levels in different GC cell lines. Tubulin served as an internal control. (**F**) DKK1 protein expression levels in CDDP-sensitive and CDDP-resistant GC cells were measured by western blotting. GAPDH served as an internal control. ** *p* < 0.01, *** *p* < 0.001, **** *p*  <  0.0001, ns: non-significance.

Given that CDDP resistance is one major reason for poor GC prognosis and one factor that leads to treatment failure, we aimed to investigate whether DKK1 participates in CDDP resistance development. First, we used two GC cell lines with acquired resistance to CDDP: HGC27/DDP and SGC7901/DDP, and their corresponding sensitive cell lines, HGC27 and SGC7901. The two acquired CDDP-resistant cell lines exhibited higher proliferative ability than the parental cell lines, as revealed by cell proliferation and Ki67 staining (Figure 2A,B). In addition, under the CDDP treatment, CDDP-sensitive cells had a higher apoptosis rate than CDDP-resistant cells, **** *p* < 0.0001 (Figure 2C). Notably, the cell viability assay with CDDP treatment demonstrated that the CDDP-resistant cells HGC27/DDP and SGC7901/DDP had higher half-maximal inhibitory concentrations (IC50) of 12.470 μM and 8.274 μM than HGC27 (3.287 μM) and SGC7901 (2.071 μM) (Figure 2D). The above data reveal that CDDP-resistant GC cells have an increased IC50, proliferation ability, and anti-apoptosis.

### 3.3. High DKK1 Expression Promotes Migration, Invasion and Proliferation in GC Cells

**Figure 3:**
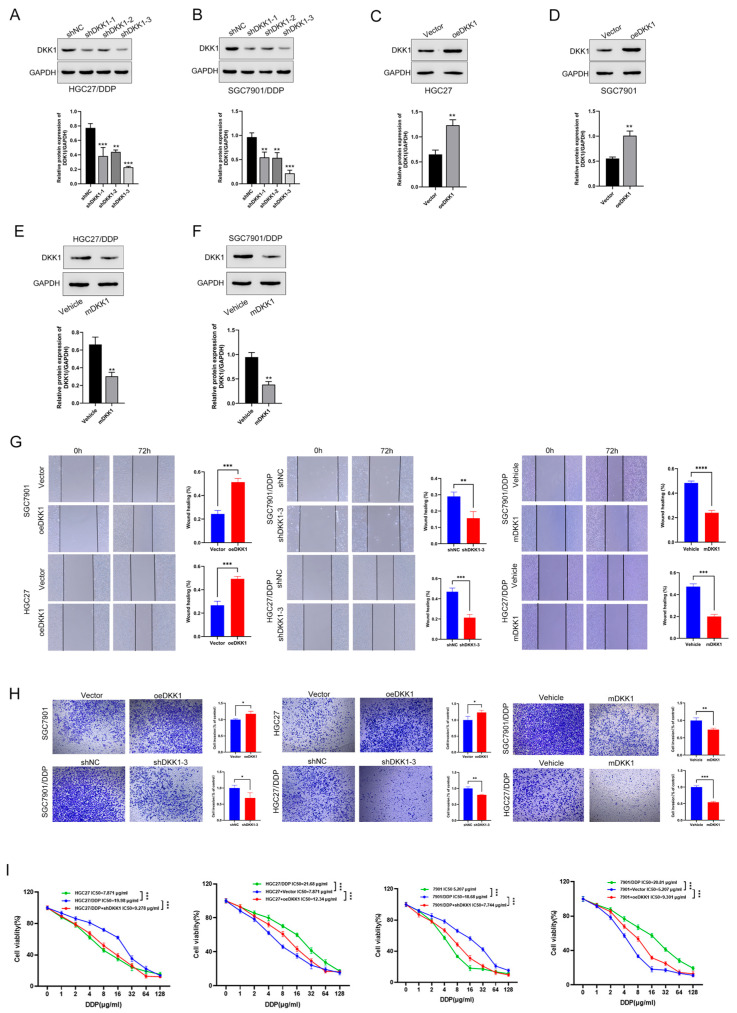
High DKK1 expression promotes cell migration, invasion, and CDDP resistance in GC cells. (**A**,**B**) CDDP-resistant cells HGC27/DDP and SGC7901/DDP cells were transfected with DKK1 shRNAs, and DKK1 expression levels were confirmed by Western blot analysis. GAPDH served as an internal control. Corresponding quantitative data for DKK1 knockdown are shown. Data are from three independent experiments. (**C**,**D**) CDDP-sensitive cells HGC27 and SGC7901 cells were transfected with DKK1 empty and expressing vectors, and then DKK1 expression levels were confirmed by Western blot analysis. GAPDH served as an internal control. Corresponding quantitative data for DKK1 knockdown are shown. Data are from three independent experiments. (**E**,**F**) CDDP-resistant HGC27/DDP and SGC7901/DDP cells were treated with mDKK1, then DKK1 expression levels were confirmed by Western blot analysis. GAPDH served as an internal control. Corresponding quantitative data for DKK1 knockdown are shown. Data are from three independent experiments. (**G**,**H**) Wound scratch (scale bar: 50 μm) and transwell assays (scale bar: 50 μm) were performed to measure cell migration and invasion in both CDDP-sensitive and CDDP-resistant cells. Data are from three independent experiments. *p* values were determined using a two-tailed unpaired Student’s *t*-test. (**I**) CCK8 assay in sensitive cells and DKK1 knockdown resistant cell lines under different concentrations of CDDP for 48 h. Data are from three independent experiments. *p* values were determined using two-way ANOVA. * *p* < 0.05, ** *p* < 0.01, *** *p* < 0.001, **** *p* < 0.0001.

To investigate the role of DKK1 in CDDP-resistant and CDDP-sensitive GC cells, we first performed western blotting to identify the expression differences in both cell lines. Interestingly, compared with that in CDDP-sensitive cells HGC27 and SGC7901 cells, DKK1 was highly expressed in CDDP-resistant cells HGC27/DDP and SGC7901/DDP cells (Figure 2E,F). Next, we constructed GC cell lines with high DKK1 expression in CDDP-sensitive cells and low DKK1 expression in CDDP-resistant cells via genetic or pharmacological approaches. DKK1 was successfully inhibited via shRNA (Figure 3A,B) and mDKK1 (Figure 3E,F) in HGC27/DDP and SGC7901/DDP cells and overexpressed in HGC27 and SGC7901 cells (Figure 3C,D). Scratch assays of proliferation and migration demonstrated that DKK1 upregulation significantly reduced the wound healing area. However, DKK1 inhibition in SGC7901/DDP and HGC27/DDP cells significantly increased the wound healing area when compared to shRNA controls (all *p* < 0.05, Figure 3G). Cell invasion experiments showed that DKK1 silencing decreased the number of invaded SGC7901/DDP and HGC27/DDP cells, while DKK1 overexpression increased the number of invaded SGC7901 and HGC27 cells (all *p* < 0.05, Figure 3H). Similar results were observed in GC cell proliferation (Figure S1). Collectively, these results showed that DKK1 enhances migration, invasion and proliferation in GC cell lines.

### 3.4. DKK1 Promotes Metastasis in GC Cell Lines and Is Associated with the EMT Phenotype

The above data show that compared to CDDP-sensitive HGC27 and SGC7901 cells, DKK1 was upregulated in CDDP-resistant HGC27/DDP and SGC7901/DDP cells. This result suggests that DKK1 may play an important role in GC metastasis. We next examined whether DKK1 expression could affect the IC50 in different cell groups treated with CDDP. As shown in Figure 3I, DKK1 gain of function in HGC27 and SGC7901 significantly increased IC50 values after CDDP treatment, thus leading to CDDP resistance. In contrast, DKK1 knockdown via a genetic method in CDDP-resistant cells (SGC7901/DDP or HGC27/DDP) regained the sensitivity to CDDP treatment, and the IC50 was downregulated accordingly in both cell lines. These results were further confirmed through anti-DKK1 antibody treatment, which served as a DKK1 inhibitor. The anti-DKK1 antibody combined with different CDDP concentrations led to CDDP IC50 values in resistant cell lines similar to those in sensitive cells (Figure 3I). 

**Figure 4:**
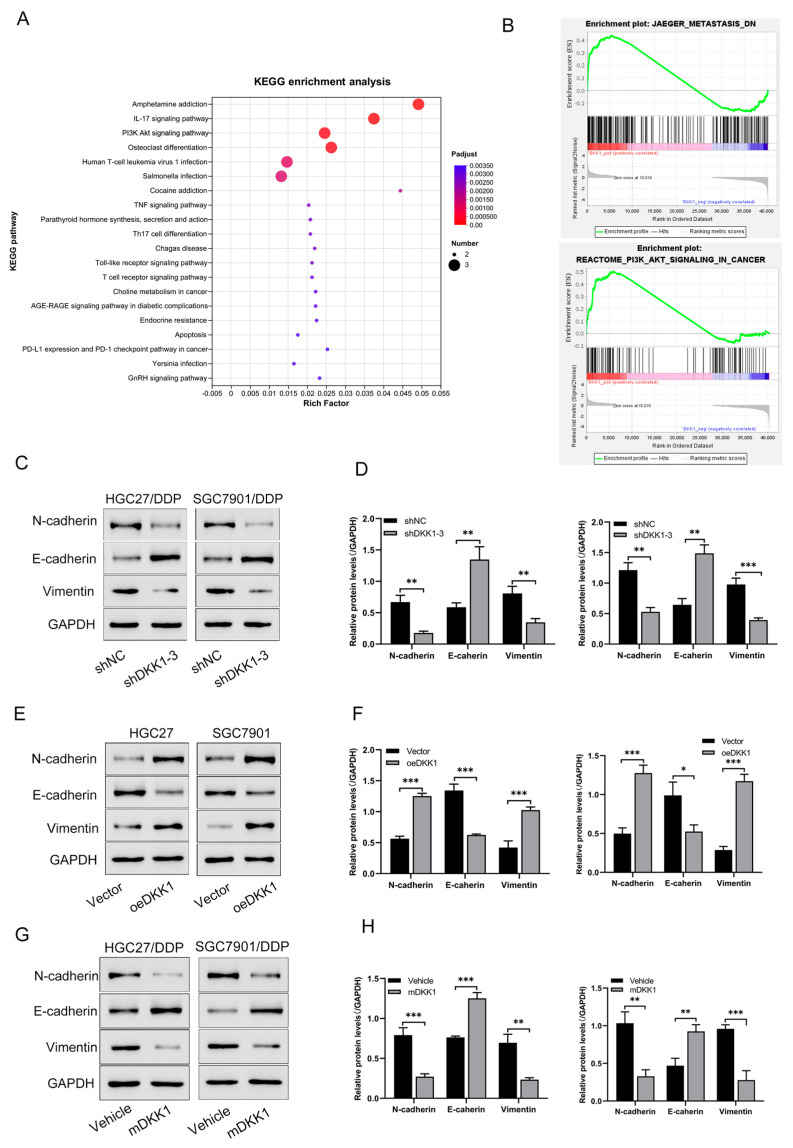
DKK1 promotes metastasis in GC cell lines and is associated with the EMT phenotype. (**A**) KEGG pathway enrichment analysis of the differentially expressed genes, top 20 related pathways expressed in HGC27/DDP and SGC7901/DDP cells with or without DKK1 knockdown were shown. (**B**) Transcriptome data were analyzed using the GSEA, and the DKK1 expression level was positively correlated with metastasis and the PI3K−AKT signaling pathway. (**C**,**D**) CDDP−resistant gastric cancer cells were transfected with DKK1 shRNA and the corresponding control shRNA for 48 h, and the expression levels of different EMT proteins (N-cadherin, E-cadherin and vimentin) were measured by western blotting. GAPDH served as an internal control. Data are from three independent experiments. Corresponding quantitative data for N-cadherin, E-cadherin and vimentin are shown. (**E**,**F**) CDDP-sensitive gastric cancer cells were transfected with empty vector and DKK1 for 48 h, and related EMT proteins (N-cadherin, E-cadherin and vimentin) were measured by western blotting. GAPDH served as an internal control. Data are from three independent experiments. Corresponding quantitative data for N-cadherin, E-cadherin and vimentin are shown. (**G**,**H**) CDDP-resistant gastric cancer cells were treated with mDKK1 for 48 h, and related EMT proteins (N-cadherin, E-cadherin and vimentin) were measured by western blotting. Data are from three independent experiments. Corresponding quantitative data for N-cadherin, E-cadherin and vimentin are shown. * *p* < 0.05, ** *p* < 0.01, *** *p* < 0.001.

In cancer biology, EMT is a significant process that tumor cells undergo and is closely related to tumor development and metastasis [23,24]. Many researchers have centered around the role of EMT in chemoresistance and found that EMT could be one of the mechanisms contributing to chemotherapy and radiotherapy resistance [22]. Does DKK1 promote CDDP resistance in an EMT-dependent manner? To test our hypothesis, signaling pathways regulated by DKK1 were analyzed via RNA-seq. According to KEGG enrichment analysis, we found the top 20 related pathways expressed in HGC27/DDP and SGC7901/DDP cells with or without DKK1 knockdown, indicating that PI3K/AKT signaling pathway may participate in CDDP resistance (Figure 4A). In addition, GSEA analysis found that the DKK1 expression level was positively correlated with metastasis (Figure 4B). As described above, CDDP-sensitive cells showed a significant increase in protein levels when transfected with the DKK1 expression plasmid, and DKK1 expression was significantly reduced when shRNA or mDKK1 was used in CDDP-resistant cells. We then measured the expression of EMT markers (N-cadherin, E-cadherin, and vimentin) when DKK1 expression was changed by shRNA or mDKK1 in human GC cell lines. 

Therefore, western blotting was used to assess the change in EMT markers in both CDDP-sensitive and CDDP-resistant cells. DKK1 knockdown in CDDP-resistant cell lines caused an increase in the protein level of E-cadherin and a reduction in the expression of N-cadherin and vimentin (Figure 4C,D). In contrast, DKK1 overexpression led to increased expression of N-cadherin and vimentin and loss of N-cadherin (Figure 4E,F). We then tested how DKK1 inhibition via an anti-DKK1 antibody affects EMT. As shown in Figure 4G,H, when CDDP-resistant cell lines were treated with mDKK1 for 48 h, similar results were observed in the shRNA group, suggesting that DKK1 inhibition via mDKK1 could alleviate the EMT process. The results indicate that increased DKK1 expression may promote EMT, which further enhances GC cell migration and metastatic ability, contributing to CDDP resistance.

### 3.5. DKK1 Promotes EMT and Proliferation by Activating the PI3K/AKT Pathway in GC Cells

**Figure 5:**
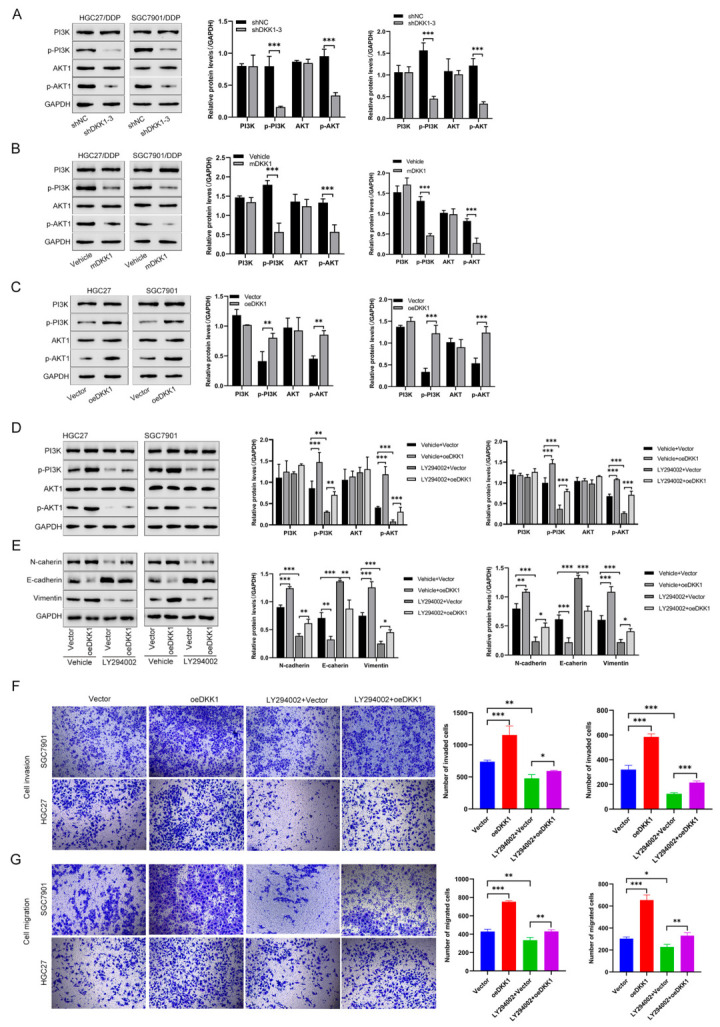
DKK1 promotes EMT by activating the PI3K/AKT pathway in GC cells. (**A**,**B**) Effects of DKK1 inhibition via shRNA or mDKK1 on the PI3K/AKT pathway in CDDP-resistant GC cells. PI3K, p-PI3K, AKT and p-AKT were detected by western blotting. GAPDH served as an internal control. Corresponding quantitative data for PI3K, p-PI3K, AKT and p-AKT are shown. *** *p* < 0.001. (**C**) HGC27 and SGC7901 cells were transfected with empty vector or DKK1, and PI3K/AKT pathway changes caused by DKK1 overexpression were measured by western blotting. GAPDH served as an internal control. Corresponding quantitative data for PI3K, p-PI3K, AKT and p-AKT are shown. ** *p* < 0.01, *** *p* < 0.001. (**D**,**E**) HGC27 and SGC7901 cells were transfected with empty vector or DKK1, and then treated with the PI3K inhibitor LY294002 for 48 h. The expression levels of PI3K, p-PI3K, AKT, p-AKT and EMT-related protein markers were detected via western blotting. (**F**,**G**) Transwell assays were performed in SGC7901 and HGC27 cells transduced with vector or DKK1 to detect the effect of DKK1 or the PI3K inhibitor LY294002 on cell invasion and migration (scale bar: 50 μm). Corresponding results are shown as the number of invasive cells per field. * *p* < 0.05, ** *p* < 0.01, *** *p* < 0.001.

Next, we further explored the molecular changes participating in DKK1-mediated EMT in GC cells. The GSEA results indicated that the DKK1 expression level was positively correlated with the PI3K-AKT pathway, and we therefore tested pathway proteins involved in the PI3K/AKT pathway. We found that DKK1 knockdown or mDKK1 treatment decreased the phosphorylation levels of PI3K and AKT (Figure 5A,B). In contrast, the elevated phosphorylation levels of PI3K and AKT were observed in DKK1 overexpressing cells (Figure 5C). To confirm whether PI3K/AKT activation played an important role in DKK1-induced EMT, the pathway inhibitor LY294002 was applied in this study to reveal the relationship between the PI3K pathway and DKK1-induced EMT. Our data demonstrated that LY294002 treatment significantly decreased the p-PI3K, p-AKT, and EMT marker levels in DKK1-overexpressing GC cells (HGC27, SGC7901) (Figure 5D). LY294002 treatment also significantly reversed the protein expression of N-cadherin, E-cadherin, and vimentin, which was induced by DKK1 (Figure 5E). Moreover, when the PI3K pathway was inhibited by LY294002 in DKK1-overexpressing GC cells, the cell invasion, migration ability and proliferation was weakened, as evidenced by Transwell assays (Figure 5F,G) and CCK8 assays (Figure S2). Together, the above data indicate that DKK1 promotes EMT and proliferation by activating the PI3K/AKT pathway.

### 3.6. DKK1 Inhibition Overcomes Cisplatin Resistance In Vitro and In Vivo

**Figure 6:**
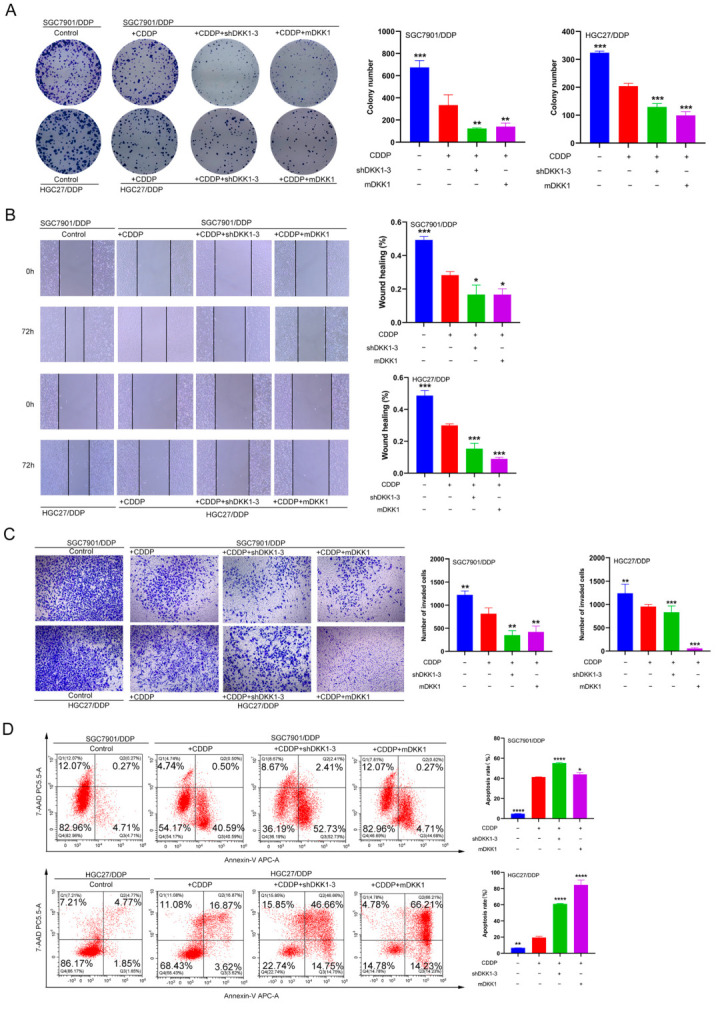
DKK1 inhibition enhances CDDP sensitivity in CDDP-resistant GC cells in vitro. (**A**) The colony formation assay (Scale bar: 50 μm) in SGC7901/DDP and HGC27/DDP cells demonstrated that DKK1 inhibition via shRNA or mDKK1 significantly suppressed cell proliferation. (**B**,**C**) Scratch migration and transwell invasion analyses were performed in HGC27/DDP and SGC7901/DDP cells (scale bar: 50 μm). The results indicated that DKK1 inhibition via shRNA or mDKK1 significantly inhibited cell migration and invasion. (**D**) Flow cytometric analysis of Annexin-V and 7-AAD staining after treatment with or without CDDP. A representative flow profile and summary of the percentage of Annexin V-positive cells are presented. * *p* < 0.05, ** *p* < 0.01, *** *p* < 0.001, **** *p* < 0.0001.

Finally, we examined the therapeutic effect of CDDP plus DKK1 inhibition treatment both in vitro and in vivo. Compared to the SGC7901/DDP or HGC27/DDP cell control group, CDDP treatment inhibited the colony formation ability of CDDP-resistant GC cells. More importantly, CDDP treatment plus DKK1 inhibition via shRNA or mDKK1 further decreased the colony number of CDDP-resistant GC cells (Figure 6A). Cell invasion and migration experiments indicated that CDDP treatment could inhibit metastatic abilities; however, DKK1 inhibition via shRNA or mDKK1 under the CDDP treatment enhanced the tendency of SGC7901/DDP and HGC27/DDP cells to lose metastatic abilities (Figure 6B,C). Furthermore, flow cytometric analysis revealed that CDDP plus shDKK1 or mDKK1 treatment achieved the highest apoptosis rate when compared to CDDP treatment alone (Figure 6D).

**Figure 7:**
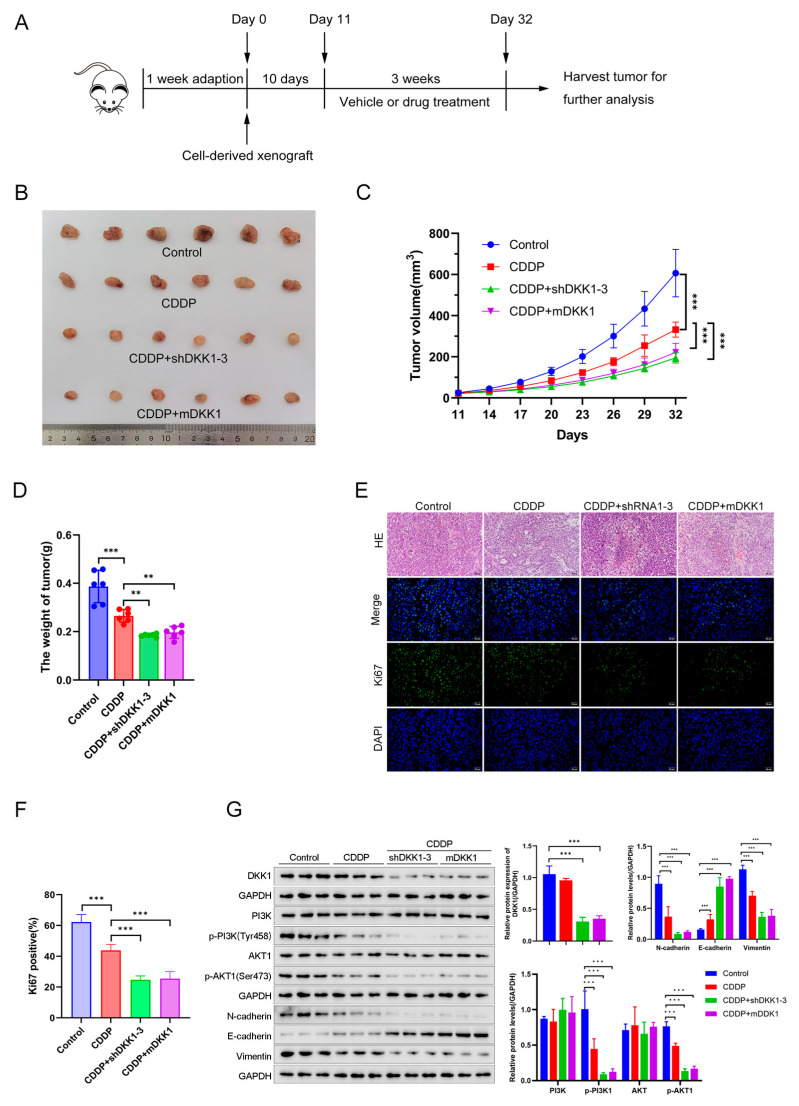
DKK1 inhibition enhances CDDP sensitivity in a CDX model. (**A**) Flowchart of construction of the cell-derived xenograft model in our study. (**B**) Images of harvested tumors in different groups on Day 32. (**C**) Tumor volume was measured every 3 days in different treatment groups (n = 6 per group). The *p* values were based on two-way ANOVA. *** *p* < 0.001. (**D**) Tumor weight was quantified in different treatment groups (n = 6 per group). ** *p* < 0.01, *** *p* < 0.001. (**E**) DKK1 inhibition was markedly associated with decreased expression of Ki67. Representative images of different tumors are shown. Scale bar: 25 μm. (**F**) Positive rate of Ki67 (*** *p* < 0.001, control vs. CDDP alone; *** *p* < 0.001, CDDP alone vs. CDDP plus DKK1 shRNA; *** *p* < 0.001, CDDP alone vs. CDDP plus mDKK1). (**G**) Protein levels of DKK1, PI3K, p-PI3K, AKT, p-AKT and EMT markers in tumor tissues were detected by western blotting. Corresponding quantitative data were shown. The *p* values were based on two-way ANOVA. *** *p* < 0.001.

Next, the animal experimental model was constructed according to the flowchart, as shown in Figure 7A. shDKK1 and shNC cells were subcutaneously inoculated into BALB/c mouse after 1 week of adaption. Each mouse was then injected intraperitoneally with either mDKK1 (20 mg/kg, twice a week, 3 weeks) or CDDP (5 mg/kg, every 3 days, 3 weeks). As shown in Figure 7B–D, the tumor sizes and weights in the CDDP treatment group were smaller than those in the corresponding nontreatment group. However, regardless of the treatment with CDDP, DKK1 knockdown further contributed to higher inhibition of tumor growth. More importantly, similar results were also observed in the group treated with mDKK1 plus CDDP. In addition, HE and Ki67 staining in tumor tissue were used to measure the tumor cell proliferation. DKK1 knockdown via shRNA or mDKK1 with CDDP treatment significantly reduced cell proliferation (Figure 7E,F) and phosphorylation levels of PI3K and AKT (Figure 7G). Hence, our in vitro and in vivo results revealed that DKK1 inhibition increases the efficacy of CDDP and could be a potential approach to reverse CDDP resistance.

## 4. Discussion

Due to the development of diagnosis and treatment methods in GC, the incidence and mortality rates of GC have declined in recent years. However, the occurrence of metastasis and resistance to chemotherapy remains a challenge for cancer treatment. Therefore, many researchers are focused on exploring the molecular changes in chemotherapy resistance models and the underlying mechanisms. In our study, we found that DKK1 was upregulated in CDDP-resistant cells HGC27/DDP and SGC7901/DDP cells compared with the corresponding CDDP-sensitive cells HGC27 and SGC7901. DKK1 belongs to the Dickkopf family, which consists of four members in vertebrates, including DKK1,2,3 and 4. Each vertebrate participates in protein–protein interactions [25]. Several studies have shown that DKK1 is an oncogene in cancers, and its aberrant expression is associated with tumor progression and poor prognosis [26,27]. DKK1 could act as a tumor suppressor or driver, depending on the tumor origin. DKK1 is a tumor suppressor in tumors arising from the ectoderm and endoderm and a promoter in mesodermal tumors. Survival data indicated that high DKK1 expression is an indicator of poor prognosis [28]. Compared with the gastric benign group and healthy group, higher serum DKK1 protein levels were observed in GC patients. Increased DKK1 levels in serum were positively correlated with TNM staging, microvascular invasion, differentiation degree, and infiltration depth [29]. Another study also analyzed the relationship between clinical parameters and DKK1 levels both in serum and tissue in GC patients [30]. Both studies revealed that serum or tissue DKK1 may serve as a potential diagnostic or prognostic marker and perhaps even a therapeutic target in GC. In addition, DKK1 also played a significant role in the tumor microenvironment (TME). DKK1 can affect different kinds of cell populations in the GC TME, including tumor-associated macrophages and T cells [31]. Inhibitors or antibodies against DKK1 may help to enhance the antitumor activity of immune cells, thus providing a novel strategy for cancer treatment.

More importantly, previous reports have demonstrated that DKK1 plays an important role in drug resistance. For instance, DKK1 was overexpressed in NSCLC cells and patients. Knockdown of DKK1 via a genetic approach sensitized NSCLC cells to CDDP [17]. Nevertheless, DKK1 was also upregulated in multiple myeloma (MM) bortezomib-resistant cells and was identified as a potential marker of a bortezomib-refractory phenotype. DKK1 could serve as a novel therapeutic target to improve the bortezomib response of MM cells [32]. However, the role of DKK1 in GC is not well studied or understood, especially its function in GC chemoresistance. Our data demonstrated that DKK1 was highly expressed in GC samples compared with adjacent normal tissues and in CDDP-resistant GC cells compared with CDDP-sensitive GC cells. In line with the prognosis data from the tissue chip, data from the GEPIA database indicated that high DKK1 expression was closely related to poor overall survival (OS) and disease-free survival (DFS) in GC. In addition, high DKK1 expression promotes cell migration, invasion, and CDDP resistance in GC cell lines. DKK1 inhibition via shRNA or mDKK1 increased the CDDP sensitivity of HGC27/DDP and SGC7901/DDP cells, while DKK1 upregulation induced CDDP resistance in HGC27 and SGC7901 cells. These data further confirmed the role of DKK1 in CDDP resistance in GC. The results from our in vivo experiment showed that DKK1 is an oncogene and effective therapeutic target in the GC treatment. Downregulation of DKK1 via shRNA or mDKK1 combined with CDDP treatment significantly inhibited tumor growth when compared with that in the group treated with CDDP alone. Our animal experiments also provide convincing evidence that DKK1 inhibition plus CDDP treatment could be a potential method to reverse CDDP resistance.

Chemotherapy is a fundamental treatment method in GC. Resistance to chemotherapy limits chemotherapeutic agents in clinical applications, and the mechanism of drug resistance requires further research. The underlying molecular and cellular mechanisms involved in CDDP resistance include increased drug efflux, increased DNA damage repair, and upregulation of anti-apoptotic factors [33,34]. EMT, an important cell process during the acquisition of tumor metastasis and drug resistance, has been widely explored in the field of cancer treatment in recent years [23,24]. According to a study recently published in Nature, the authors found that skin squamous cell carcinoma was more likely to undergo EMT during tumorigenesis and further contribute to chemotherapy resistance. The results from cell and animal experiments demonstrated that EMT cancer cells are insensitive to various anticancer therapies [22]. EMT has also been proven to be a key regulator in colorectal cancer metastasis and chemoresistance, and its inhibition could enable cancer cells to become sensitive to chemotherapy again [35]. Whether DKK1 can regulate EMT-mediated chemotherapy resistance in GC is unknown. In this study, we demonstrated that DKK1 contributes to CDDP resistance by regulating EMT in GC cells. Transwell and scratch assays of proliferation and migration revealed that DKK1 overexpression in CDDP-sensitive cell lines could promote cell migration and invasion, while DKK1 knockdown in CDDP-resistant GC cells could reverse the above tendency. Previous research reported that drug-resistant cancer cells were more likely to undergo EMT [36,37]. Similarly, we found that enhanced mesenchymal properties tend to occur in CDDP-resistant GC cells (high DKK1 expression) when compared to CDDP-sensitive GC cells (low DKK1 expression), as evidenced by the downregulation of E-cadherin, and the upregulation of N-cadherin and vimentin. DKK1 overexpression in CDDP-sensitive cells enhanced EMT, and DKK1 knockdown in CDDP-resistant cells significantly inhibited EMT, which was proven by the changes in EMT-related markers. Our data indicate that DKK1 plays an important role in the metastasis and EMT of CDDP-resistant cells. 

The PI3K/AKT signaling pathway is commonly deregulated in various cancers [38]. It can regulate cell survival, proliferation, and metastasis and is an important pathway in transferring signals from extracellular to intracellular [39,40]. More importantly, previous reports confirmed that the PI3K/AKT signaling pathway is closely related to the CDDP resistance, and inhibition of AKT expression can effectively reverse the CDDP resistance in cancer cells [41,42,43]. Thus, we first analyzed our transcriptome data using the GSEA and KEGG enrichment and found that the DKK1 expression level was positively correlated with metastasis and the PI3K-AKT pathway. We also investigated whether the PI3K/AKT pathway was involved in DKK1-mediated CDDP resistance in GC cells. Our results demonstrated that DKK1 knockdown through shRNA or anti-DKK1 antibody caused decreased expression of p-PI3K and p-AKT in CDDP-resistant GC cells. In contrast, DKK1 overexpression increased the expression level of p-PI3K and p-AKT. Accordingly, the above signaling pathway tendency was reversed when treating DKK1 overexpression cells (HGC27/DKK1, SGC7901/DKK1) with LY294002. Therefore, DKK1 promotes EMT and CDDP resistance via activation of the PI3K/AKT pathway. 

The interaction between DKK1 and cytoskeleton-associated protein 4 (CKAP4) offers a window into a deeper understanding of tumor biology and the development of new therapeutic strategies. CKAP4 was initially believed to be a protein related to the cellular cytoskeleton and associated with cellular morphology and movement [44]. However, with further research, it was found that CKAP4 plays a crucial role in many biological processes, especially in the initiation, progression, and metastasis of tumors. CKAP4 has been identified as a type II transmembrane protein capable of binding with various ligands, including DKK1 [45]. The binding DKK1 and CKAP4 can trigger downstream signaling pathways, including PI3K/AKT and MAPK1/3, thereby influencing cellular functions and promoting the proliferation, migration, and invasion of tumor cells [46]. There have been several studies on the DKK1/CKAP4 axis in gastrointestinal tumors. For instance, in esophageal squamous cell carcinoma (ESCC), the DKK1 and CKAP4 interaction is considered one of the key mechanisms driving ESCC cell proliferation and tumor formation [47]. Moreover, the co-expression of DKK1 and CKAP4 is associated with hepatocellular carcinoma (HCC) invasion and poor prognosis. The expression of DKK1 and CKAP4 is essential for tumor formation in vivo, suggesting that CKAP4 is a potential molecular target for HCC therapy. A strong inhibitory effect on HCC cell proliferation was observed when anti-CKAP4 Ab and lenvatinib were combined. This research further emphasized the therapeutic potential of the DKK1/CKAP4 axis [48]. *H. pylori* infection is a well-known factor that contributes to the genesis and development of GC. Recent evidence has shown that DKK1 transcription could be induced by *H. pylori* infection and further lead to gastric tumorigenesis via activation of the CKAP4/PI3K/AKT/mTOR pathway [49]. Herein, identifying small molecule drugs or antibodies blocking the DKK1/CKAP4 axis could be an important aspect of GC treatment.

In the current study, we provided another perspective that DKK1 plays a critical role in GC chemoresistance and that the downstream PI3K/AKT signaling pathway is activated by DKK1 in CDDP-resistant GC cell lines. Genetic inhibition via shRNA or pharmacological inhibition via mDKK1 mitigates the EMT process and restores CDDP sensitivity both in vivo and in vitro. However, does the DKK1/CKAP4 axis participate in GC chemoresistance? Serum DKK1 level differences in CDDP-sensitive and CDDP-resistant GC patients remain to be elucidated. These are limitations in this study and will be priorities in our future research.

## 5. Conclusions

In conclusion, the current study revealed that DKK1 was highly expressed in the GC CDDP-resistant GC cells and GC tissues, and its increased expression was associated with poor prognosis in GC patients. DKK1 could activate the PI3K/AKT pathway and affect EMT, further contributing to CDDP resistance. Genetic knockdown and pharmacological inhibition of DKK1 recovered CDDP sensitivity both in vitro and in vivo. Therefore, our study highlights the potential of targeted inhibition of DKK1 to reverse CDDP resistance and alleviate metastatic properties in GC.

## Figures and Tables

**Table 1:** Correlations between DKK1 expression and clinicopathologic features of GC patients.

Clinical Parameters		Cases	DKK1 Expression		χ^2^	*p* Value
			Low	High		
Sex	Male	45	30	15	0.615	0.433
	Female	21	16	5		
Age	≤60	40	27	13	0.232	0.630
	>60	26	19	7		
Tumor size	≤3 cm	27	15	12	4.326	0.038
	>3 cm	39	31	8		
Pathologic_T	T1–2	19	16	3	2.661	0.103
	T3–4	47	30	17		
Pathologic_N	N0	21	18	3	3.741	0.053
	N1–3	45	28	17		
Pathologic_M	M0	62	43	19	0.000	1.000
	M1	4	3	1		
TNM stage	I–II	29	24	5	4.179	0.041
	III–IV	37	22	15		
Differentiation	I–II/II	16	12	4	0.167	0.683
	II–III/III	36	25	11		

**Table 2:** Results of univariate analysis for overall survival in GC tissue microarray.

Variable	Univariate Analysis	
	*p*-Value	HR(95% CI)
Sex (male vs. female)	0.421	1.345 (0.653–2.771)
Age (≤60 vs. >60)	0.300	1.403 (0.739–2.661)
Pathologic_T (T1/T2 vs. T3/T4)	0.005	3.864 (1.501–9.943)
Pathologic_N (N0 vs. N1–3)	0.002	4.618 (1.795–11.884)
Pathologic_M (M0 vs. M1)	0.069	2.704 (0.926–7.892)
TNM (I/II vs. III/IV)	0.003	3.061 (1.478–6.341)
Tumor_size (≤3 cm vs. 3 cm)	0.050	2.017 (0.999–4.074)
Differentiation (I–II/II vs. II–II/III)	0.793	0.899 (0.406–1.989)

## Data Availability

The bioinformatical data involved in this study can be found here: GEPIA (Gene Expression Profiling Interactive Analysis; http://gepia.cancer-pku.cn/ (accessed on 3 March 2022)) and TIMER (http://timer.comp-genomics.org/timer/ (accessed on 3 March 2022)). Additional information required can be made available upon reasonable request to the corresponding author.

## Supplementary Materials

The following supporting information can be downloaded at: https://www.mdpi.com/article/10.3390/cancers15194756/s1, Figure S1: DKK1 promotes proliferation in GC cells; Figure S2: DKK1 mediates GC cell proliferation through the PI3K/AKT1 pathway.

## References

[1] Sung H., Ferlay J., Siegel R.L., Laversanne M., Soerjomataram I., Jemal A., Bray F. (2021). Global Cancer Statistics 2020: GLOBOCAN Estimates of Incidence and Mortality Worldwide for 36 Cancers in 185 Countries. CA Cancer J. Clin..

[2] Sexton R.E., Al Hallak M.N., Diab M., Azmi A.S. (2020). Gastric cancer: A comprehensive review of current and future treatment strategies. Cancer Metastasis Rev..

[3] Wang F.H., Zhang X.T., Li Y.F., Tang L., Qu X.J., Ying J.E., Zhang J., Sun L.Y., Lin R.B., Qiu H. (2021). The Chinese Society of Clinical Oncology (CSCO): Clinical guidelines for the diagnosis and treatment of gastric cancer, 2021. Cancer Commun..

[4] Siddik Z.H. (2003). Cisplatin: Mode of cytotoxic action and molecular basis of resistance. Oncogene.

[5] Galanski M.S. (2006). Recent Developments in the Field of Anticancer Platinum Complexes. Recent Pat. Anti-Cancer Drug Discov..

[6] Amable L. (2016). Cisplatin resistance and opportunities for precision medicine. Pharmacol. Res..

[7] Huang D., Savage S.R., Calinawan A.P., Lin C., Zhang B., Wang P., Starr T.K., Birrer M.J., Paulovich A.G. (2021). A highly annotated database of genes associated with platinum resistance in cancer. Oncogene.

[8] Jiang H., Zhang Z., Yu Y., Chu H.Y., Yu S., Yao S., Zhang G., Zhang B.T. (2022). Drug Discovery of DKK1 Inhibitors. Front. Pharmacol..

[9] Li J., Gong W., Li X., Wan R., Mo F., Zhang Z., Huang P., Hu Z., Lai Z., Lu X. (2018). Recent Progress of Wnt Pathway Inhibitor Dickkopf-1 in Liver Cancer. J. Nanosci. Nanotechnol..

[10] Yao L., Zhang D., Zhao X., Sun B., Liu Y., Gu Q., Zhang Y., Zhao X., Che N., Zheng Y. (2016). Dickkopf-1-promoted vasculogenic mimicry in non-small cell lung cancer is associated with EMT and development of a cancer stem-like cell phenotype. J. Cell. Mol. Med..

[11] Xu W.H., Liu Z.B., Yang C., Qin W., Shao Z.M. (2012). Expression of dickkopf-1 and beta-catenin related to the prognosis of breast cancer patients with triple negative phenotype. PLoS ONE.

[12] Jiang T., Huang L., Zhang S. (2013). DKK-1 in serum as a clinical and prognostic factor in patients with cervical cancer. Int. J. Biol. Markers.

[13] Rachner T.D., Thiele S., Gobel A., Browne A., Fuessel S., Erdmann K., Wirth M.P., Frohner M., Todenhofer T., Muders M.H. (2014). High serum levels of Dickkopf-1 are associated with a poor prognosis in prostate cancer patients. BMC Cancer.

[14] Liu Z., Sun B., Qi L., Li Y., Zhao X., Zhang D., Zhang Y. (2015). Dickkopf-1 expression is down-regulated during the colorectal adenoma-carcinoma sequence and correlates with reduced microvessel density and VEGF expression. Histopathology.

[15] Mikheev A.M., Mikheeva S.A., Rostomily R., Zarbl H. (2007). Dickkopf-1 activates cell death in MDA-MB435 melanoma cells. Biochem. Biophys. Res. Commun..

[16] Xu H., Wu J., Chen B., Li M., Tian Y., He M., Xue J., Wang J., Bai S., Sharma A. (2014). Serum Dickkopf-1 (DKK1) is significantly lower in patients with lung cancer but is rapidly normalized after treatment. Am. J. Transl. Res..

[17] Salim H., Zong D., Haag P., Novak M., Mork B., Lewensohn R., Lundholm L., Viktorsson K. (2015). DKK1 is a potential novel mediator of cisplatin-refractoriness in non-small cell lung cancer cell lines. BMC Cancer.

[18] Chaffer C.L., Weinberg R.A. (2011). A perspective on cancer cell metastasis. Science.

[19] Loh C.Y., Chai J.Y., Tang T.F., Wong W.F., Sethi G., Shanmugam M.K., Chong P.P., Looi C.Y. (2019). The E-Cadherin and N-Cadherin Switch in Epithelial-to-Mesenchymal Transition: Signaling, Therapeutic Implications, and Challenges. Cells.

[20] Satelli A., Li S. (2011). Vimentin in cancer and its potential as a molecular target for cancer therapy. Cell. Mol. Life Sci. CMLS.

[21] Hong S.K., Lee H., Kwon O.S., Song N.Y., Lee H.J., Kang S., Kim J.H., Kim M., Kim W., Cha H.J. (2018). Large-scale pharmacogenomics based drug discovery for ITGB3 dependent chemoresistance in mesenchymal lung cancer. Mol. Cancer.

[22] Debaugnies M., Rodriguez-Acebes S., Blondeau J., Parent M.A., Zocco M., Song Y., de Maertelaer V., Moers V., Latil M., Dubois C. (2023). RHOJ controls EMT-associated resistance to chemotherapy. Nature.

[23] Brabletz T., Kalluri R., Nieto M.A., Weinberg R.A. (2018). EMT in cancer. Nat. Rev. Cancer.

[24] Shibue T., Weinberg R.A. (2017). EMT, CSCs, and drug resistance: The mechanistic link and clinical implications. Nat. Rev. Clin. Oncol..

[25] Chu H.Y., Chen Z., Wang L., Zhang Z.K., Tan X., Liu S., Zhang B.T., Lu A., Yu Y., Zhang G. (2021). Dickkopf-1: A Promising Target for Cancer Immunotherapy. Front. Immunol..

[26] Zhu G., Song J., Chen W., Yuan D., Wang W., Chen X., Liu H., Su H., Zhu J. (2021). Expression and Role of Dickkopf-1 (Dkk1) in Tumors: From the Cells to the Patients. Cancer Manag. Res..

[27] Fezza M., Moussa M., Aoun R., Haber R., Hilal G. (2019). DKK1 promotes hepatocellular carcinoma inflammation, migration and invasion: Implication of TGF-beta1. PLoS ONE.

[28] Doucet D., Brubaker C., Turner D., Gregory C.A. (2023). Factors affecting the role of canonical Wnt inhibitor Dickkopf-1 in cancer progression. Front. Oncol..

[29] Zhuang G.F., Tan Y., Zeng J.T., Zhang J.W., Tang J., Zeng S.P., Qin X. (2015). Expression of serum Dickkopf-1 in gastric cancer patients. Asian Pac. J. Trop. Med..

[30] Lee H.S., Lee H.E., Park D.J., Kim H.H., Kim W.H., Park K.U. (2012). Clinical significance of serum and tissue Dickkopf-1 levels in patients with gastric cancer. Clin. Chim. Acta Int. J. Clin. Chem..

[31] Shi T., Zhang Y., Wang Y., Song X., Wang H., Zhou X., Liang K., Luo Y., Che K., Wang X. (2022). DKK1 Promotes Tumor Immune Evasion and Impedes Anti-PD-1 Treatment by Inducing Immunosuppressive Macrophages in Gastric Cancer. Cancer Immunol. Res..

[32] Zhang Q., Gong W., Wu H., Wang J., Jin Q., Lin C., Xu S., Bao W., Wang Y., Wu J. (2021). DKK1 suppresses WWP2 to enhance bortezomib resistance in multiple myeloma via regulating GLI2 ubiquitination. Carcinogenesis.

[33] Marin J.J., Al-Abdulla R., Lozano E., Briz O., Bujanda L., Banales J.M., Macias R.I. (2016). Mechanisms of Resistance to Chemotherapy in Gastric Cancer. Anticancer Agents Med. Chem..

[34] Navaei Z.N., Khalili-Tanha G., Zangouei A.S., Abbaszadegan M.R., Moghbeli M. (2021). PI3K/AKT signaling pathway as a critical regulator of Cisplatin response in tumor cells. Oncol. Res..

[35] Zhang J., Miller Z., Musich P.R., Thomas A.E., Yao Z.Q., Xie Q., Howe P.H., Jiang Y. (2020). DSTYK Promotes Metastasis and Chemoresistance via EMT in Colorectal Cancer. Front. Pharmacol..

[36] Ge L., Li D.S., Chen F., Feng J.D., Li B., Wang T.J. (2017). TAZ overexpression is associated with epithelial-mesenchymal transition in cisplatin-resistant gastric cancer cells. Int. J. Oncol..

[37] Duan X., Luo M., Li J., Shen Z., Xie K. (2022). Overcoming therapeutic resistance to platinum-based drugs by targeting Epithelial-Mesenchymal transition. Front. Oncol..

[38] Carnero A., Blanco-Aparicio C., Renner O., Link W., Leal J.F. (2008). The PTEN/PI3K/AKT signalling pathway in cancer, therapeutic implications. Curr. Cancer Drug Targets.

[39] Brozovic A., Osmak M. (2007). Activation of mitogen-activated protein kinases by cisplatin and their role in cisplatin-resistance. Cancer Lett..

[40] Corradetti M.N., Guan K.L. (2006). Upstream of the mammalian target of rapamycin: Do all roads pass through mTOR?. Oncogene.

[41] Liu L.Z., Zhou X.D., Qian G., Shi X., Fang J., Jiang B.H. (2007). AKT1 amplification regulates cisplatin resistance in human lung cancer cells through the mammalian target of rapamycin/p70S6K1 pathway. Cancer Res..

[42] Cai Y., Tan X., Liu J., Shen Y., Wu D., Ren M., Huang P., Yu D. (2014). Inhibition of PI3K/Akt/mTOR signaling pathway enhances the sensitivity of the SKOV3/DDP ovarian cancer cell line to cisplatin in vitro. Chin. J. Cancer Res. Chung-Kuo Yen Cheng Yen Chiu.

[43] Gohr K., Hamacher A., Engelke L.H., Kassack M.U. (2017). Inhibition of PI3K/Akt/mTOR overcomes cisplatin resistance in the triple negative breast cancer cell line HCC38. BMC Cancer.

[44] Suchitha G.P., Balaya R.D.A., Raju R., Prasad T.S.K., Dagamajalu S. (2023). A network map of cytoskeleton-associated protein 4 (CKAP4) mediated signaling pathway in cancer. J. Cell Commun. Signal..

[45] Kikuchi A., Fumoto K., Kimura H. (2017). The Dickkopf1-cytoskeleton-associated protein 4 axis creates a novel signalling pathway and may represent a molecular target for cancer therapy. Br. J. Pharmacol..

[46] Kimura H., Fumoto K., Shojima K., Nojima S., Osugi Y., Tomihara H., Eguchi H., Shintani Y., Endo H., Inoue M. (2016). CKAP4 is a Dickkopf1 receptor and is involved in tumor progression. J. Clin. Investig..

[47] Shinno N., Kimura H., Sada R., Takiguchi S., Mori M., Fumoto K., Doki Y., Kikuchi A. (2018). Activation of the Dickkopf1-CKAP4 pathway is associated with poor prognosis of esophageal cancer and anti-CKAP4 antibody may be a new therapeutic drug. Oncogene.

[48] Iguchi K., Sada R., Matsumoto S., Kimura H., Zen Y.H., Akita M., Gon H., Fukumoto T., Kikuchi A. (2023). DKK1-CKAP4 signal axis promotes hepatocellular carcinoma aggressiveness. Cancer Sci..

[49] Luo M., Chen Y.J., Xie Y., Wang Q.R., Xiang Y.N., Long N.Y., Yang W.X., Zhao Y., Zhou J.J. (2022). Dickkopf-related protein 1/cytoskeleton-associated protein 4 signaling activation by Helicobacter pylori-induced activator protein-1 promotes gastric tumorigenesis via the PI3K/AKT/mTOR pathway. World J. Gastroenterol..

