# Supplementary material for: DKK1 Promotes Epithelial–Mesenchymal Transition and Cisplatin Resistance in Gastric Cancer via Activation of the PI3K/AKT Pathway
Source: Cancers (Basel). 2023 Sep 27;15(19):4756. doi: 10.3390/cancers15194756 (PMC10571993; doi:10.3390/cancers15194756)
Supplement: Supplementary file 1 [file cancers-15-04756-s001.zip › cancers-2595807-supplementary.pdf]

# Supplementary Materials

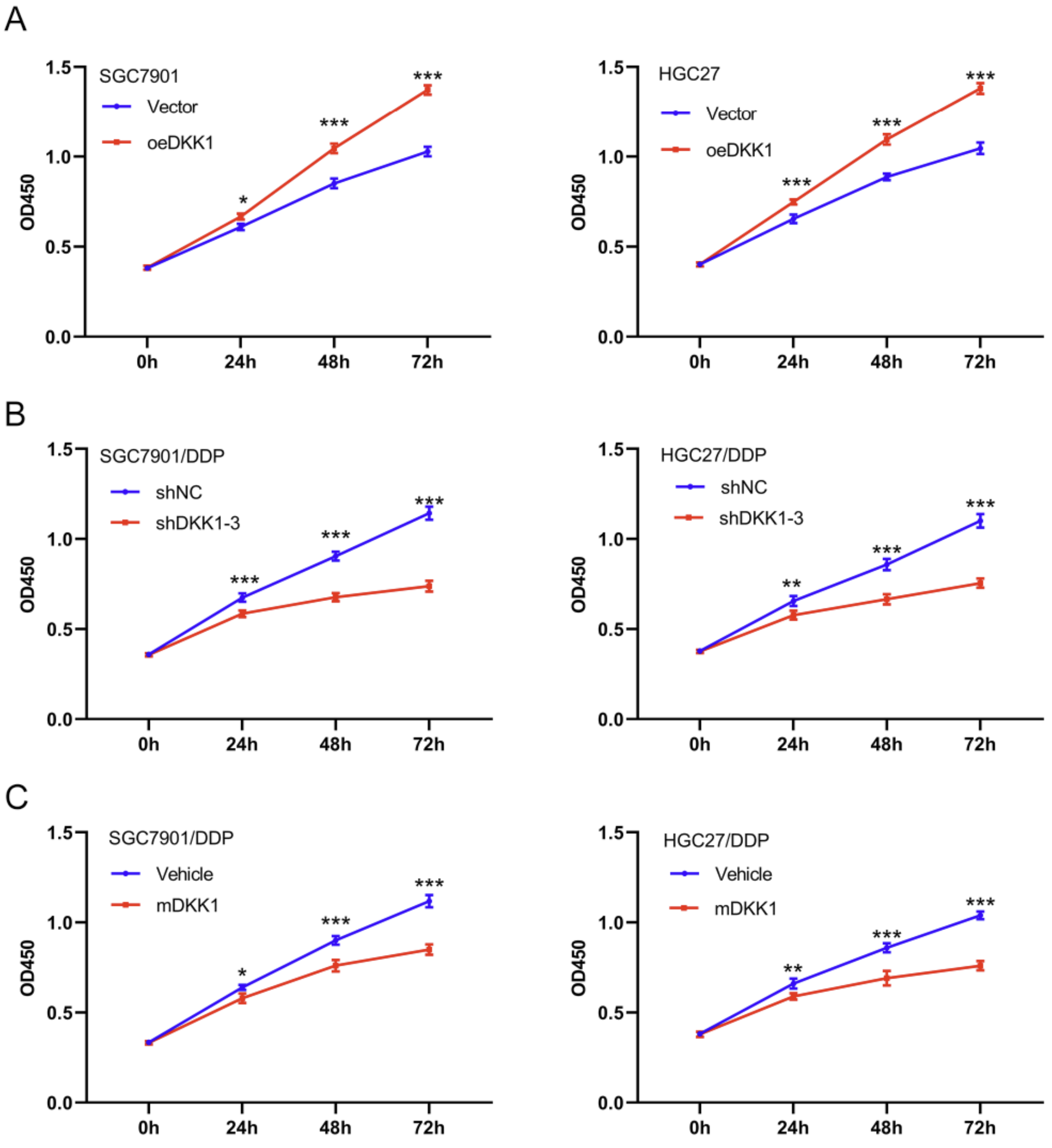


**Supplementary Figure S1.**

**DKK1 promotes proliferation in GC cells.**

**(A)** CCK-8 assay was performed to detect the proliferation of SGC7901 and HGC27 cells transfected with DKK1 overexpressing plasmid. **(B)** CCK-8 assay was performed to detect the proliferation of SGC7901/DDP and HGC27/DDP cells transfected with DKK1 shRNA. **(C)** CCK-8 assay was performed to detect the proliferation of SGC7901/DDP and HGC27/DDP cells treated with mDKK1. p is based on Student's t test. *$p < 0.05$, **$p < 0.01$, ***$p < 0.001$.

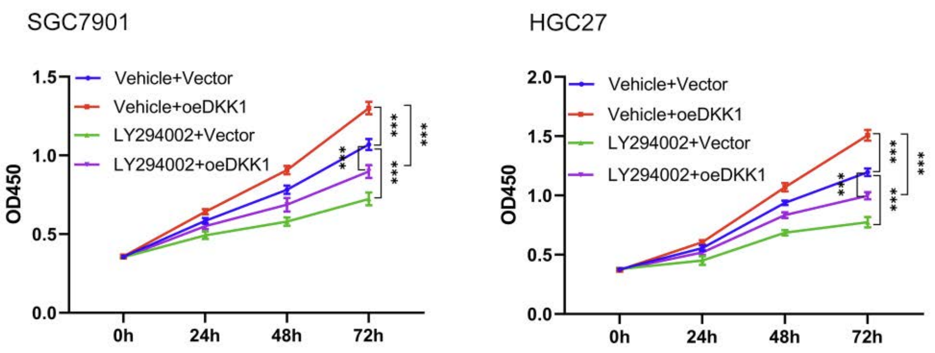


**Supplementary Figure S2.**

**DKK1 mediates GC cell proliferation through the PI3K/AKT1 pathway.**

CCK-8 assay was performed to detect the proliferation of SGC7901 and HGC27 cells transfected with DKK1 overexpressing plasmid or/and treated with PI3K/AKT signaling pathway inhibitor LY294002. p is based on two-way ANOVA. ***$p < 0.001$.

Figure 2F

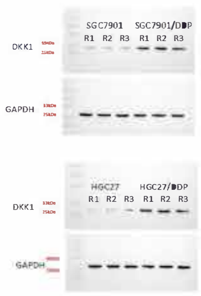

Figure 3A-F

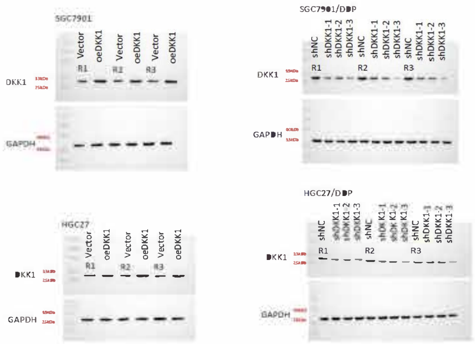

Figure 4C,E,G

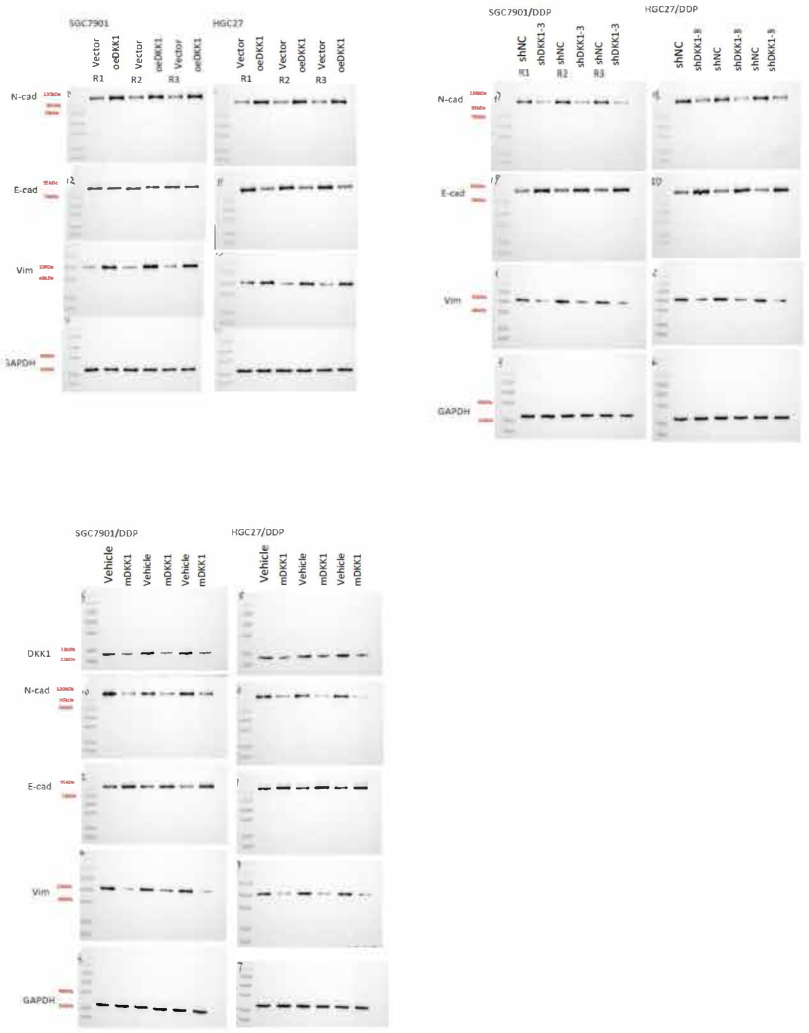

Figure 5A-C

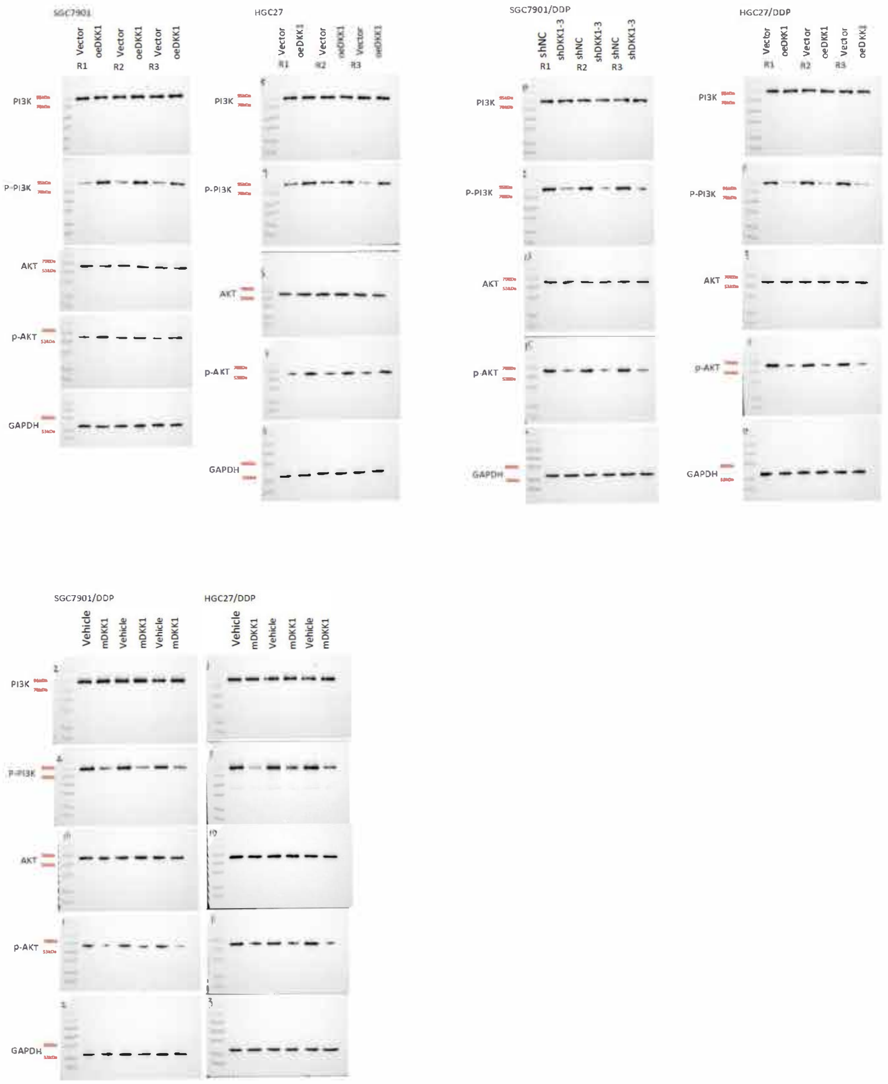


Figure 5D,E

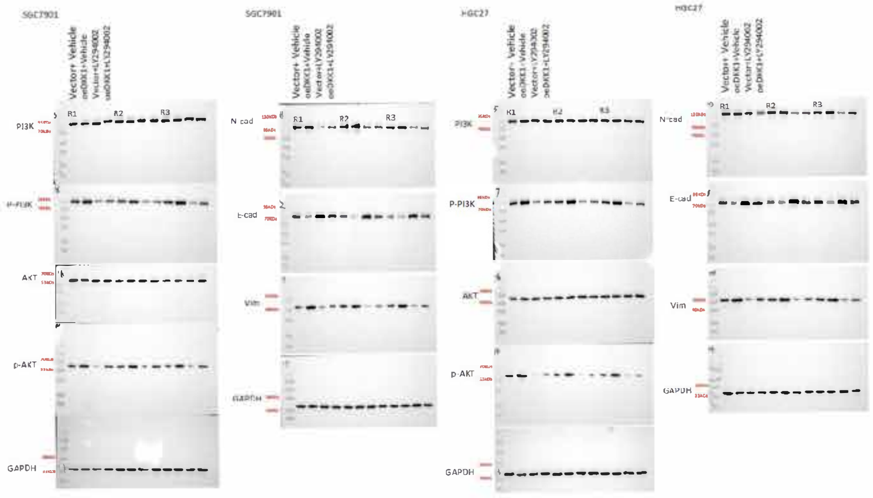

Figure 7G

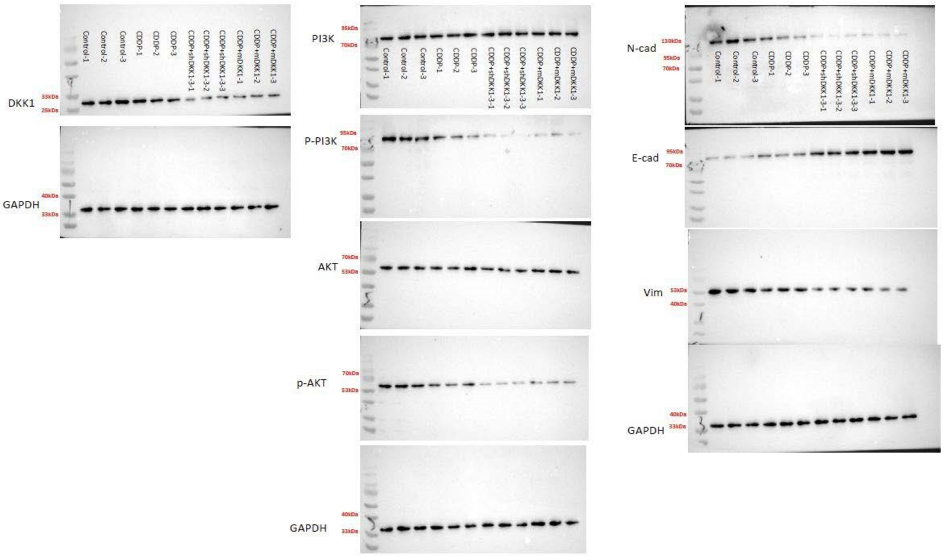


The original western blot figures.
